# Neonatal Diabetes: Two Cases with Isolated Pancreas Agenesis due to Homozygous PTF1A Enhancer Mutations and One with Developmental Delay, Epilepsy, and Neonatal Diabetes Syndrome due to KCNJ11 Mutation

**DOI:** 10.4274/jcrpe.5162

**Published:** 2018-05-18

**Authors:** Olcay Evliyaoğlu, Oya Ercan, Emel Ataloğlu, Ümit Zübarioğlu, Bahar Özcabı, Aydilek Dağdeviren, Hande Erdoğan, Elisa De Franco, Sian Ellard

**Affiliations:** 1 İstanbul University Cerrahpaşa Faculty of Medicine, Department of Pediatric Endocrinology, İstanbul, Turkey; 2 University of Health Science, Haseki Training and Research Hospital, Newborn Intensive Unit, İstanbul, Turkey; 3 Şişli Hamidiye Etfal Training and Research Hospital, Newborn Intensive Unit, İstanbul, Turkey; 4 University of Exeter Medical School, Institute of Biomedical and Clinical Science, Exeter, United Kingdom

**Keywords:** Neonatal diabetes, PTF1A, pancreas agenesis, KCNJ11

## Abstract

Neonatal diabetes mellitus is a rare form of monogenic diabetes which is diagnosed in the first six months of life. Here we report three patients with neonatal diabetes; two with isolated pancreas agenesis due to mutations in the pancreas-specific transcription factor 1A (PTF1A) enhancer and one with developmental delay, epilepsy, and neonatal diabetes (DEND) syndrome, due to a KCNJ11 mutation. The two cases with mutations in the distal enhancer of PTF1A had a homozygous g.23508363A>G and a homozygous g.23508437A>G mutation respectively. Previous functional analyses showed that these mutations can decrease expression of PTF1A which is involved in pancreas development. Both patients were born small for gestational age to consanguineous parents. Both were treated with insulin and pancreatic enzymes. One of these patients’ fathers was also homozygous for the PTF1A mutation, whilst his partner and the parents of the other patient were heterozygous carriers. In the case with DEND sydrome, a previosly reported heterozygous KCNJ11 mutation, p.Cys166Tyr (c.497G>A), was identified. This patient was born to nonconsanguineous parents with normal birth weight. The majority of neonatal diabetes patients with KCNJ11 mutations will respond to sulphonylurea treatment. Therefore Glibenclamide, an oral antidiabetic of the sulphonylurea group, was started. This treatment regimen relatively improved blood glucose levels and neurological symptoms in the short term. Because we could not follow the patient in the long term, we are not able to draw conclusions about the efficacy of the treatment. Although neonatal diabetes mellitus can be diagnosed clinically, genetic analysis is important since it is a guide for the treatment and for prognosis.

## What is already known on this topic?

Permanent neonatal diabetes can be due to either disruption of pancreas development or of insulin secretion. Pancreas-specific transcription factor 1A is a transcription factor which is required for normal development of the pancreas. Mutations at this site cause pancreas agenesis. Closure of ATP sensitive potassium (K) channels depolarize the cell membrane and subsequently open voltage-dependent Ca channels that trigger release of stored insulin granules. KCNJ11 activating mutations result in the ATP-sensitive K channel remaining open and disrupting insulin secretion.

## What this study adds?

In two patients with neonatal diabetes and exocrine pancreas insufficiency homozygous g. 23508363>G and g.23508437A>G mutations in the distal pancreas-specific transcription factor 1A enhancer were identified. A previously reported heterozygous KCNJ11 missense mutation, p.C166Y, was identified in the third patient who had developmental delay, epilepsy, and neonatal diabetes syndrome. In patients with neonatal diabetes genetic causes should be investigated not just for finding the underlying cause but also for planning treatment.

## Introduction

Neonatal diabetes mellitus (NDM) is a rare form of monogenic diabetes that can be caused by mutations in different genes and presents in the first six months of life (1). There are two main clinical groups; transient NDM (TNDM) and permanent NDM (PNDM). TNDM is a developmental insulin production disorder that resolves spontaneously postnatally. PNDM does not go into remission. The underlying genetic defect can be found in most of the patients with TNDM. The majority of cases with TNDM are due to methylation defects in the imprinted region on chromosome 6q24; these can be either paternal uniparental disomy, paternal duplication, or defective methylation of the maternal allele (2). 

PNDM is a genetically heterogeneous disorder due to mutations in 23 different genes described to date: *KCNJ11*, *ABCC8*, *FOXP3*, *GCK*, *PDX1*, pancreas-specific transcription factor 1A *(PTF1A)*, *EIF2AK3*, *SLC2A2*, *GATA6*, *GATA4*, *SLC19A2*, *WFS1*, *NEUROD1*, *NEUROG3*, *RFX6*, *LRBA*, *NKX2-2*, *MNX1*, *IER3IP1*, *INS*, *STAT3*, *GLIS3* and *HNF1B* (3,4,5,6,7,8,9). These mutations can either compromise insulin secretion, disturb pancreas or islet cell development or result in autoimmune destruction of the beta cells. Genes associated with pancreatic agenesis are *PDX1, PTF1A, RFX6, HNF1B *and* GATA6* (5,6). Disruption of pancreas development leads to exocrine as well as endocine pancreatic insufficiency. Mutations in the genes encoding the ATP-sensivitive potassium channel (K_ATP_) subunits, *KCNJ11 (Kir6.2), ABCC8 *sulphonylurea receptor 1 *(SUR1) *and *INS* (insulin) compromise insulin secretion by affecting the mechanisms involved in insulin secretion (10,11,12,13,14). 

In this report we describe three patients with neonatal diabetes. Two of these cases had isolated pancreatic agenesis due to mutations in a distal enhancer of the *PTF1A* gene. The third patient had normal pancreatic development and additional neurological symptoms due to a mutation in the *KCNJ11* gene.

## Case Reports

### Case 1

This patient was a female infant born to consanguineous parents (first cousins). She was born at 37 weeks gestation by normal vaginal delivery with a birth weight of 1900 g. After birth she was followed in the neonatal intensive care unit for respiratory distress and hyperglycemia. She was treated with subcutaneous (SC) regular insulin. She has two healthy siblings. There was no family history of diabetes. 

At the age of one month she was referred to our clinic because of uncontrolled high blood glucose levels. On admission her body weight was 2330 g [-3.05 standard deviation score (SDS)], height was 47 cm (-2.67 SDS), head circumference was 35 cm (-1.72 SDS). Her physical examination was normal, except for hip dysplasia. 

Laboratory tests revealed a venous glucose level of 354 mg/dL with glycosuria. She did not have ketonuria or acidosis. Serum C-peptide level was 0.01ng/mL (normal range: 0.9-4.3 ng/mL), hemoglobulin A1c (HbA1c) was 7.3% (normal range: 4.8-6%) and diabetes autoantibody tests [anti glutamic acid decarboxylase (GAD), islet cell antibodies (ICA), insulinoma antigen-2 (IA2)] were negative. Hb level was 9.5 mg/dL, and mean corpuscular volume (MCV) was 85.1 fL (normal range: 81-99 fL). The peripheral blood smear showed no signs of megaloblastic anemia. Serum folic acid, thiamine and vitamin B12 levels were normal. Serum thyroid hormones were within normal limits [thyrotrophin-stimulating hormone (TSH): 1.75 mIU/L, fT4: 1.06 ng/dL]. Renal and hepatic function tests were all within normal ranges. The patient was diagnosed as a case of neonatal diabetes and insulin regimen was changed from SC NPH insulin, with which blood glucose levels could not be stabilized, to detemir insulin, with rapid acting insulin adjustment when needed. But this regimen was also not successful in controlling the blood glucose levels. Finally detemir insulin was replaced with glargine insulin (1.0 U/day) that achieved more stable blood glucose levels. Humalog insulin (0.5 U/dose) was added when needed. Insulin doses were adjusted according to blood glucose levels. The patient also had episodes of significant diarrhea and stool tests revealed malabsorption. Abdominal ultrasonography revealed normal liver and kidneys, but the pancreas could not be visualized. Her echocardiography was normal. For exocrine pancreas insufficiency, enzyme replacement therapy was added to her treatment to which she responded well (Table 1). At her last visit she was 3.5 years old, her body weight was 13.9 kg (-0.64 SDS), height was 92.3 cm (-1.57 SDS), head circumference was 48 cm (-1.08 SDS) with normal mental and motor development. Her glucose regulation was in acceptable ranges with a HbA1c level of 7.3 %.

### DNA Sequencing and Genetic Analysis

**Figure 1:**
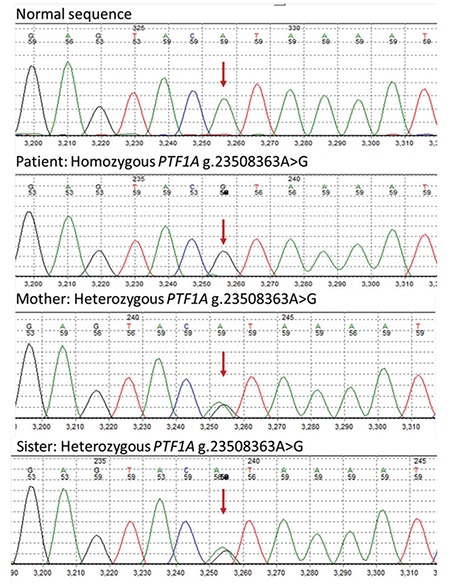
A homozygous g.23508363A>G mutation within distal enhancer of *PTF1* was identified in the first case. Mother and sister were heterozygous for the same mutation

A homozygous g.23508363A>G mutation affecting a highly conserved nucleotide within a previously identified distal enhancer of PTF1A was identified (Figure 1). Previous functional analyses showed that this mutation disrupts enhancer activity and is likely to result in decreased PTF1A expression during pancreatic development (15). This result confirms a diagnosis of neonatal diabetes and exocrine pancreatic insufficiency due to a recessive PTF1A mutation. Her mother and sister were heterozygous for the same mutation. Her brother did not carry the mutation. Father’s sample was not available for testing.

### Case 2

The second case was a female infant born to consanguineous parents (first cousins). She was born at 37 weeks of gestation with a birth weight of 1520 g by C-section delivery due to oligohydramnios and intrauterine growth restriction. She was followed in the neonatal intensive care unit for hyperglycemia and was treated with SC regular insulin. She was a first child with no siblings. There was no family history of diabetes.

She was referred to our clinic for glucose regulation when she was 44 days old. Her body weight was 1980 g (-3.93 SDS), height was 43 cm (-5.06), head circumference was 32 cm (-4.75). Her physical examination was normal.

Laboratory tests showed venous glucose was 300 mg/dL accompanied by glycosuria. She did not have ketonuria or acidosis. Her serum C-peptide level was 0.01 ng/mL (normal range: 0.9-4.3 ng/mL), HbA1c was 7.4% (normal range: 4.8-6%) and diabetes autoantibody tests (antiGAD, ICA, IA2) were negative. Hb level was 8 mg/dL, and MCV was 85 fL (normal range: 81-99 fL). The pheripheral blood smear showed no signs of megalobastic anemia. Serum folic acid, thiamine and vitamin B12 levels were normal. Thyroid function was normal (TSH: 5.8 mIU/L, fT4: 0.9 ng/dL). Renal and hepatic function tests were all within normal ranges. The patient was diagnosed with neonatal diabetes. We started glargine insulin (1 U/day). Humalog insulin (0.5 U /dose) was added when needed. Her mother was eager to use insulin pump therapy. Thus she was trained for it and a better glycemic control was achieved by continous SC insulin pump treatment. She was fed breast milk, thus her baseline insulin dose was 0.125 U/hour which was modified according to her blood glucose levels.  Her bolus insulin dose was 0.5 U which was again modified according to her blood glucose levels.

Abdominal ultrasonography and MRI images failed to visualize the pancreas whilst liver and kidneys appeared normal. Her stool tests were significant for malabsorption. Her echocardiography revealed patent foramen ovale, thin patent ductus arteriosus and peripheral pulmonary stenosis. Enzyme replacement treatment for pancreatic insufficiency was added to her treatment regimen and she responded well.

At her last visit she was 4 months old, her body weight was 4500 g (-2.64 SDS), height was 53 cm (-3.92), head circumference was 38 cm (-2.68 SDS) with normal mental and motor development. Both her family and she were adapted to insulin pump therapy (Table 1). Her blood glucose levels were within appropriate levels with an HbA1c level of 7.1%.

### DNA Sequencing and Genetic Analysis

**Figure 2:**
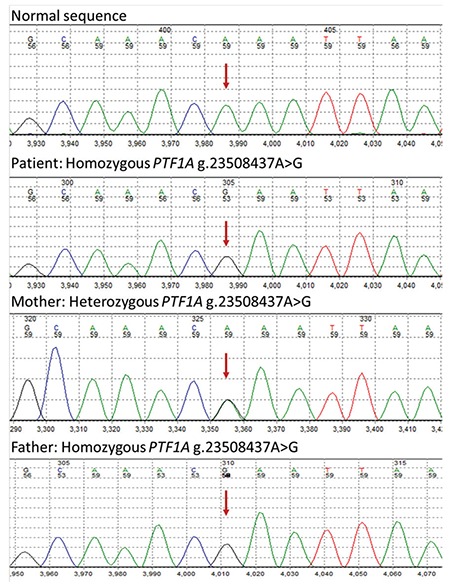
A homozygous g.23508437A>G mutation was identified within the distal enhancer of the *PTF1A* gene in the second case. For the same mutation mother was heterozygous, father was also homozygous

A homozygous g.23508437A>G mutation was identified within the distal enhancer of the *PTF1A* gene which is known to affect a highly conserved nucleotide (Figure 2). Previous functional analysis had shown that this mutation disrupts enhancer activity and is likely to result in decreased PTF1A expression during pancreatic development (15). This result confirms a diagnosis of neonatal diabetes and exocrine pancreatic insufficiency due to a recessive PTF1A mutation. The patient’s mother was heterozygous for the mutation whilst the unaffected father was also homozygous. One patient with a homozygous g.23508437A>G mutation who developed diabetes in adulthood has been previously reported (15). Her father is therefore at increased risk of developing diabetes and annual monitoring of his HbA1c was recommended. The risk for this couple’s next pregnancy to be affected with neonatal diabetes is 1 in 2.

### Case 3

The third case was a male infant born to non-consanguineous parents. He was born at 35 weeks gestation by spontaneous vaginal delivery with a birth weight of 3400 g. His seizures, manifesting as arm movements, started when he was one month old which then progressed as tonic clonic convulsions. He was the first and only child of his family and there was no family history of diabetes.

High blood glucose levels and failure to thrive were the reasons for referral to our clinic at the age of three months. On admission body weight was 4460 g (-2.24 SDS), height was 62.5 cm (0.44), head circumference was 40 cm (-0.81 SDS). His physical examination revealed hypotonia and decreased muscle strength of all four extremities. He was not following with his eyes.

Laboratory measurements of venous blood glucose level was 600 mg/dL with glycosuria, which was not accompanied by ketonuria or acidosis. Serum C-peptide level was 0.72 ng/mL (normal range: 0.9-4.3 ng/mL), HbA1c was 11.4% (normal range: 4.8-6%) and diabetes autoantibody tests (antiGAD, ICA, IA2) were negative. Hb level was 11.4 mg/dL, and MCV was 84 fL (normal range: 81-99 fL). The peripheral blood smear showed no signs of megalobastic anemia. Serum folic acid, thiamine and vitamin B12 levels were normal. Serum thyroid hormone measurements were within normal limits (TSH: 1.75 mIU/L, fT4: 1.06 ng/dL). Renal and hepatic function tests were all within normal ranges. In accordance with our previous experience, we started glargine insulin. Humalog insulin was added when needed according to the patient’s blood glucose levels. His tonic clonic convulsions continued and were unrelated to blood sugar levels. An electro‐encephalogram was performed and phenobarbital was started. Developmental delay, epilepsy and neonatal diabetes suggested developmental delay, epilepsy, and neonatal diabetes (DEND) syndrome. Genetic testing detected a heterozygous missense mutation, c.497G>A p.C166Y, in KCNJ11 which had been previously reported. Glibenclamide, an oral antidiabetic belonging to the sulfonylurea group, was started according to the protocol provided by the Exeter team (available at http://www.diabetesgenes.org/content/genetic-testing-neonatal-diabetes). Glibenclamide dose was gradually increased while insulin dose was decreased (Table 1). With this treatment regimen his blood sugar levels were well controlled and a relative improvement (normal muscle tone, eye contact) in his neurological status was observed at the seven month follow-up visit. At his last visit he was 10 months old, his body weight was 6190 g (-3.39 SDS), height was 74 cm (-0.29 SDS), head circumferance was 44 cm (-1.65 SDS). He was on glibenclamide and insulin treament at doses of 10mg/day and 4 U/day (2 U glargine and 2 U Humalog insulins) respectively.

### DNA Sequencing and Genetic Analysis

**Figure 3:**
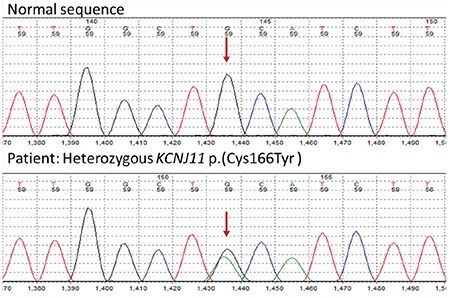
A heterozygous missense mutation, p.C166Y within *KCNJ11* gene was identified in the third casev

The patient was heterozygous for a previosly reported KCNJ11 missense mutation, p.C166Y (Figure 3). The p.C166Y mutation has been reported previously in patients with DEND syndrome. This mutation is predicted to be pathogenic and the result confirmed a diagnosis of neonatal diabetes, epilepsy and developmental delay due to a mutation in the Kir6.2 subunit of the K_ATP_ channel (16). The inform consent was taken from all the patients’ parents for publication.

## Discussion

Here we report three cases with neonatal diabetes caused by three different mutations, two homozygous mutations in the PTF1A enhancer and one heterozygous mutation in the *KCNJ11* gene. Their common finding was hyperglycemia before six months of age. Cases 1 and 2 were born small for gestational age and had exocrine pancreatic insufficiency. Case 3 was born with an appropriate for gestational age birth weight and had neurological symptoms. Although all were diagnosed with neonatal diabetes, their clinical findings suggested different modes of disease development. 

Heterozygous activating mutations in KCNJ11, encoding the Kir6.2 subunit of the K_ATP_, are common cause of neonatal diabetes and have been reported as being the reason in 30-58 % of neonatal diabetes cases (12,16,17,18,19). However, in populations with a high incidence of consanguineous marriages, homozygous mutations causing neonatal diabetes appear to be more common (20,21).

Exocrine pancreas insufficieny in the first two cases suggested pancreas agenesis. Thus, homozygous g. 23508363>G and g.23508437A>G mutations in the distal PTF1A enhancer were identified. Spatiotemporally regulated expression of transcription factors is important for cell fate specification and organogenesis (22). PTF1A is a transcription factor that is required for the formation of the exocrine pancreas and the correct spatial organization of the endocrine pancreas in mice (23). In mice models, PTF1A inactivation reverted pancreatic cells to intestinal cells, suggesting its function as a switch between pancreatic and intestinal cell fates (24) and PTF1A dose reduction resulted in pancreatic hypoplasia and insufficient insulin secretion in a dosage dependent manner (22). Furthermore, in humans coding mutations in PTF1A were shown to cause neonatal diabetes due to pancreas agenesis (25). Weedon et al (15) have identified six different recessive mutations in a downstream enhancer of PTF1A in 10 families with isolated pancreas agenesis (15). This region acts as a developmental enhancer of PTF1A and the mutations abolish enhancer activity. It was interesting to find the same homozygous mutation in the distal PTF1A enhancer in the healthy father of the second case. However, a patient who developed diabetes in adulthood with a homozygous g.23508437A>G mutation has been previously reported (15). The reason for this situation is not known clearly. But it is well known that there is no genotype phenotype correalation in many genetic diseases. There should be more factors that effect gene functions other than the gene mutation it self. 

Neurological symptoms in Case 3 suggested DEND syndrome and a previously reported heterozygous KCNJ11 missense mutation, p.C166Y, was identified. ATP sensitive K_ATP_ channels couple cell metabolism to membrane excitability in various cell types, including neurons, pancreatic beta cells, endocrine and muscle cells. The archetypal K_ATP_ channel is an octameric complex of Kir6.2 and either SUR1 or SUR2 subunits. Pancreatic beta cells and many neurons involve SUR1, muscle cells involve SUR2. Four Kir6.2 subunits form the channel pore, and each is associated with a SUR subunit that regulates channel gating (26). In pancreatic beta cells, ATP-potassium channels regulate glucose-induced insulin secretion. In the unstimulated state, the beta cell K_ATP_ channels are open. Following the uptake and metabolism of glucose, intracellular ATP/ADP ratio increases which results in closure of K_ATP_ channels, depolarization of the cell membrane, and subsequent opening of voltage-dependent calcium (Ca) channels. Increase in cytosolic Ca concentration triggers the release of stored insulin granules. KCNJ11 activating mutations result in the K_ATP_ channel remaining open and insulin secretion is therefore disrupted. Pancreas development is normal and diabetes is due to impaired insulin secretion. This channel is important in numerous sites such as neurological cells. Thus, mutations can result not only in diabetes but can also lead to neurological disorders.

*KCNJ11* gene mutation can be treated with an oral antidiabetic agent, sulfonylurea, which can close the K_ATP_ channel and induce insulin secretion (27). This treatment has been shown to also improve neurological symptoms (28,29,30,31,32,33). However, in patients with mutations resulting in severe DEND syndrome, sulfonylurea treatment is often ineffective (10,16,27,34). Although in a Brazilian patient having the same mutation as our third patient (p.C166Y mutation in the *KCNJ11* gene) sulfonylurea treatment was unsuccesful in controlling blood glucose levels and neurological symptoms (35), we observed relative improvement in both blood glucose levels and neurological symptoms in the short term. Nevertheless, we cannot comment on treatment success because we were not able to follow this patient in the long term. 

Although neonatal diabetes is a rare disorder, it should be promptly evaluated for additional clinical findings. Clinical findings can be a clue for choosing the genetic tests. Genetic testing is important because it not only reveals the underlying mechanism for the disorder but will also guide treatment and follow up of the patients.

## Figures and Tables

**Table 1:**
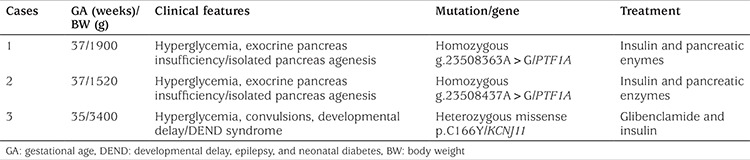
Clinical features, genetic defects and treatment modules of the patients
